# Supplementary material for: A common mechanism allows selective targeting of GluN2B subunit-containing N-methyl-D-aspartate receptors
Source: Commun Biol. 2019 Nov 15;2:420. doi: 10.1038/s42003-019-0645-6 (PMC6858350; doi:10.1038/s42003-019-0645-6)
Supplement: Supplementary file 1 — Supplementary Material [file 42003_2019_645_MOESM1_ESM.pdf]

Supplementary Figures

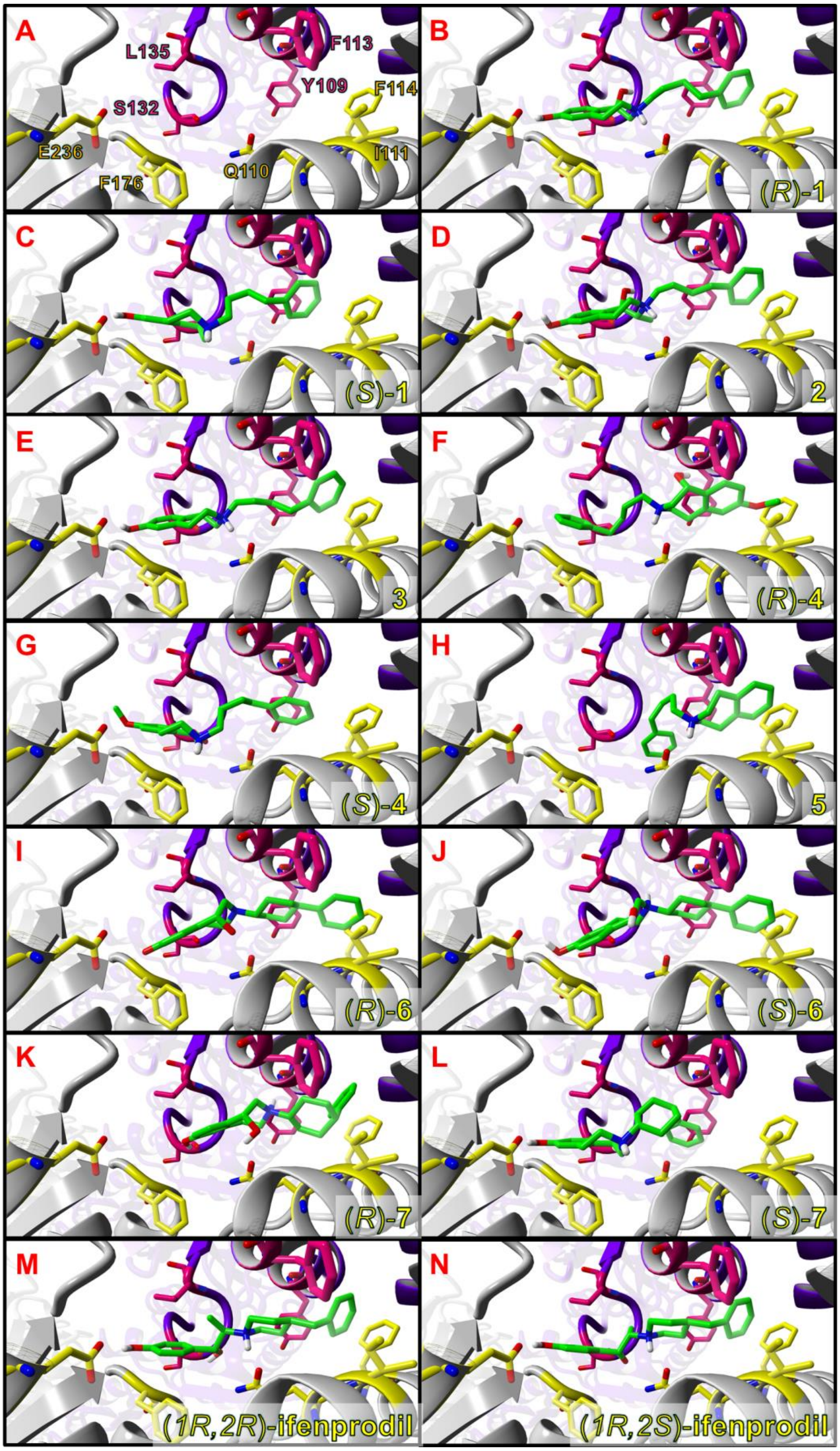

Supplementary Figure 1: Selected docking conformations of ifenprodil and 3-benzazepines 1-7

Docking conformations with highest binding energy for all tested compounds at GluN1-1a (purple)/GluN2B (gray) ATD interface (PDB 4PE5). Mutated residues are shown in magenta (GluN1-1a) and yellow (GluN2B) (Supplementary Figure 1A). compounds are CPK-colored with C-Atoms in green (Supplementary Figures 1B-1N).

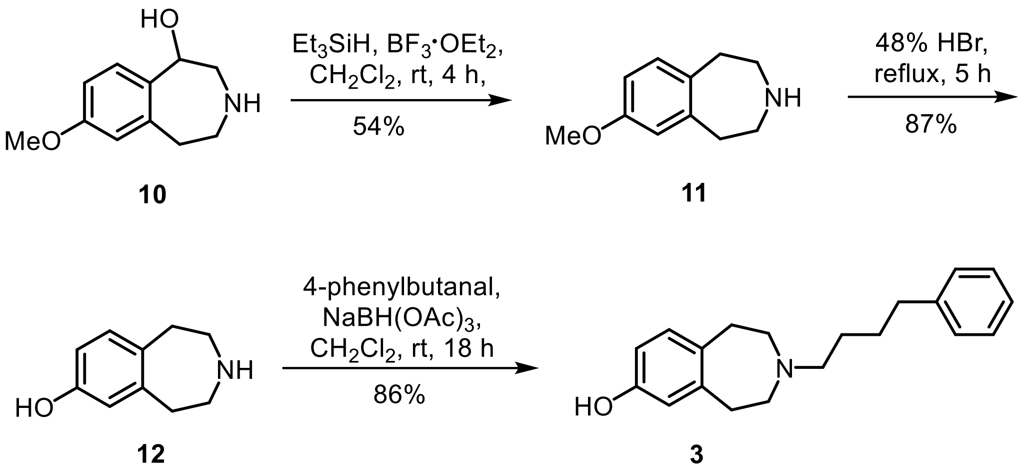

Supplementary Figure 2: Synthesis of compound 3

3-Benzazepin-7-ol **3** was prepared in three steps starting with dehydroxylation of commercially available 7-methoxy-2,3,4,5-tetrahydro-1*H*-3-benzazepin-1-ol (**10**) and subsequent cleavage of methyl ether and reductive alkylation with 4-phenylbutanal. Detailed synthesis information is given in supplementary methods.

Supplementary tables

**Supplementary Table 1 selectivity of 3-Benzazepines compared to ifenprodil**				
compound	GluN2A<br>% ± SEM	GluN2B<br>% ± SEM	GluN2C<br>% ± SEM	GluN2D<br>% ± SEM
ifenprodil	5 ± 1 (5)	91 ± 1 (3)	9 ± 1 (5)	24 ± 4 (5)
(*R*)-**1**	4 ± 1 (5; ns)	95 ± 1 (3; ns)	8 ± 1 (5; ns)	18 ± 3 (5; ns)
(*S*)-**1**	6 ± 2 (5; ns)	93 ± 1 (3; ns)	19 ± 2 (5; ns)	21 ± 3 (5; ns)
**2**	15 ± 5 (5; ns)	95 ± 1 (3; ns)	56 ± 2 (5; ***)	55 ± 2 (5; ***)

Inhibition at 10 µM (*R*)-**1**, (*S*)-**1** and **2** was evaluated by TEVC-measurements at oocytes expressing NMDARs with different GluN2 subunits and compared to 10 µM ifenprodil. Results are given as percentage of ion current inhibition ± SEM. Number of independent oocytes are given in brackets. Significance was analyzed by one-way-ANOVA and post-hoc mean comparison Tukey test ($p > 0.05$ = ns; $p < 0.05$ = *; $p < 0.01$ = **; $p < 0.001$ = ***).

Supplementary Table 2 inhibitory activity of ifenprodil

GluN1-1a	GluN2B	IC_{50} ± SE [nM]	A2 ± SE [%]	p	IC_{50} shift factor	n
WT	WT	264 ± 27	94 ± 2	0.97	-	24
Y109S	WT	447 ± 70	74 ± 2	0.88	1.7	18
F113S	WT	436 ± 78	72 ± 3	1.09	1.7	18
S132A	WT	690 ± 94	89 ± 3	0.79	2.6	18
L135N	WT	917 ± 143	76 ± 3	0.99	3.5	18
WT	Q110A	643 ± 159	98 ± 5	0.64	2.4	15
WT	I111A	1623 ± 611	68 ± 7	0.90	6.1	15
WT	F114A	52584 ± 20310	94*	0.63	199.2	12
WT	F176A	23413 ± 2557	94*	0.87	88.7	12
WT	F176Y	642 ± 99	94*	0.57	2.4	18
WT	E236A	945 ± 187	88 ± 5	1.18	3.6	15

Activity of ifenprodil at wildtype (WT) and mutated GluN1-1a/GluN2B receptors evaluated in TEVC experiments. Experimental data were fitted to a logistic sigmoidal equation with flexible or fixed-to-wildtype (*) maximum inhibition (A2) and flexible slope (p). IC_{50} and A2 are given with standard error of the fit (SE). IC_{50} shift factor displays ratio of mutant IC_{50} to wildtype IC_{50}, while n represents the number of independent oocytes, which were used to calculate the dose-response curve.

**Supplementary Table 3 inhibitory activity of (*R*)-1**						
GluN1-1a	GluN2B	IC_{50} ± SE [nM]	A2 ± SE [%]	p	IC_{50} shift factor	n
WT	WT	53 ± 3	93 ± 1	1.11	-	24
Y109S	WT	20 ± 2	92 ± 2	1.42	0.4	18
F113S	WT	117 ± 48	77 ± 5	0.50	2.2	18
S132A	WT	106 ± 14	92 ± 2	0.74	2.0	18
L135N	WT	293 ± 74	72 ± 3	0.66	5.5	18
WT	Q110A	393 ± 24	91 ± 1	2.18	7.4	15
WT	I111A	137 ± 31	66 ± 3	0.92	2.6	15
WT	F114A	4085 ± 423	93*	1.35	77.1	12
WT	F176A	104786 ± 41041	93*	0.40	1977.1	12
WT	F176Y	107 ± 20	93*	0.82	2.0	18
WT	E236A	194 ± 30	85 ± 1	1.32	3.7	18

Activity of (*R*)-**1** at wildtype (WT) and mutated GluN1-1a/GluN2B receptors evaluated in TEVC experiments. Experimental data were fitted to a logistic sigmoidal equation with flexible or fixed-to-wildtype (*) maximum inhibition (A2) and flexible slope (p). IC_{50} and A2 are given with standard error of the fit (SE). IC_{50} shift factor displays ratio of mutant IC_{50} to wildtype IC_{50}, while n represents the number of independent oocytes, which were used to calculate the dose-response curve.

**Supplementary Table 4 inhibitory activity of (*S*)-1**						
GluN1-1a	GluN2B	IC_{50} ± SE [nM]	A2 ± SE [%]	p	IC_{50} shift factor	n
WT	WT	206 ± 23	95 ± 2	0.98	-	24
Y109S	WT	764 ± 189	79 ± 4	0.85	3.7	18
F113S	WT	756 ± 137	89 ± 4	0.87	3.7	18
S132A	WT	691 ± 44	89 ± 1	1.16	3.4	18
L135N	WT	3657 ± 1182	68 ± 7	0.98	17.8	18
WT	Q110A	243 ± 34	94 ± 2	1.27	1.2	15
WT	I111A	1564 ± 504	73 ± 6	0.89	7.6	15
WT	F114A	38879 ± 5282	95*	1.26	188.7	12
WT	F176A	15527 ± 1787	95*	1.00	75.4	12
WT	F176Y	261 ± 25	95*	0.82	1.3	18
WT	E236A	1981 ± 597	96 ± 8	0.99	9.6	15

Activity of (*S*)-**1** at wildtype (WT) and mutated GluN1-1a/GluN2B receptors evaluated in TEVC experiments. Experimental data were fitted to a logistic sigmoidal equation with flexible or fixed-to-wildtype (*) maximum inhibition (A2) and flexible slope (p). IC_{50} and A2 are given with standard error of the fit (SE). IC_{50} shift factor displays ratio of mutant IC_{50} to wildtype IC_{50}, while n represents the number of independent oocytes, which were used to calculate the dose-response curve.

**Supplementary Table 5 inhibitory activity of 2**						
GluN1-1a	GluN2B	IC_{50} ± SEM [nM]	A2 ± SE [%]	p	IC_{50} shift factor	n
WT	WT	91 ± 7	97 ± 2	1.08	-	24
Y109S	WT	140 ± 19	96 ± 2	0.86	1.5	18
F113S	WT	76 ± 17	100 ± 3	0.57	0.8	18
S132A	WT	387 ± 57	100 ± 3	0.78	4.3	18
L135N	WT	835 ± 184	100 ± 5	0.72	9.2	18
WT	Q110A	130 ± 6	95 ± 1	1.79	1.4	15
WT	I111A	380 ± 82	96 ± 4	0.86	4.2	15
WT	F114A	667 ± 90	97*	1.03	7.3	15
WT	F176A	1810 ± 127	97*	0.94	19.9	12
WT	F176Y	84 ± 9	97*	1.00	0.9	18
WT	E236A	108 ± 16	95 ± 3	1.09	1.2	15

Activity of **2** at wildtype (WT) and mutated GluN1-1a/GluN2B receptors evaluated in TEVC experiments. Experimental data were fitted to a logistic sigmoidal equation with flexible or fixed-to-wildtype (*) maximum inhibition (A2) and flexible slope (p). IC_{50} and A2 are given with standard error of the fit (SE). IC_{50} shift factor displays ratio of mutant IC_{50} to wildtype IC_{50}, while n represents the number of independent oocytes, which were used to calculate the dose-response curve.

**Supplementary Table 6 binding energy and dissociation constants**		
Ligand	Calculated binding energy [kcal/mol]	Calculated dissociation constant [pM]
(*R*)-**1**	11.88	1970
(*S*)-**1**	12.18	1190
**2**	12.29	985
**3**	11.77	2350
(*R*)-**4**	11.95	1750
(*S*)-**4**	12.25	1050
**5**	11.38	4580
(*R*)-**6**	12.09	1370
(*S*)-**6**	12.04	1500
(*R*)-**7**	12.72	477
(*S*)-**7**	12.94	327
(1*R*,2*R*)-ifenprodil	12.73	470
(1*R*,2*S*)-ifenprodil	13.45	138

Calculated binding energies and dissociation constants from docking experiments for ifenprodil and **1**-**7**. Values were taken from the best docking conformation of 50 docking runs per ligand.

Supplementary Methods

Chemistry, general

Unless otherwise noted, moisture sensitive reactions were conducted under dry nitrogen. CH_2Cl_2 was distilled over CaH_2. Thin layer chromatography (tlc): Silica gel 60 F_{254} plates (Merck). Flash chromatography (fc): Silica gel 60, 40–64 µm (Merck); parentheses include: diameter of the column (d), length of the column (l), fraction size (v), eluent. Melting point: Melting point apparatus Mettler Toledo MP50 Melting Point System, uncorrected. MS: microTOF-Q II (Bruker Daltonics); APCI, atmospheric pressure chemical ionization. IR: FT-IR spectrophotometer MIRacle 10 (Shimadzu) equipped with ATR technique. Nuclear magnetic resonance (NMR) spectra were recorded on Agilent 600-MR (600 MHz for ^{1}H, 151 MHz for ^{13}C) or Agilent 400-MR spectrometer (400 MHz for ^{1}H, 101 MHz for ^{13}C); δ in ppm related to tetramethylsilane and measured referring to $CHCl_3$ (δ = 7.26 ppm (^{1}H NMR) and δ = 77.2 ppm (^{13}C NMR)) and CHD_2OD (δ = 3.31 ppm (^{1}H NMR) and δ = 49.0 ppm (^{13}C NMR)); coupling constants are given with 0.5 Hz resolution; the assignments of ^{13}C and ^{1}H NMR signals were supported by 2-D NMR techniques where necessary. HPLC: Merck Hitachi Equipment; UV detector: L-7400; autosampler: L-7200; pump: L-7100; degasser: L-7614; column: LiChrospher® 60 RP-select B (5 µm); LiChroCART® 250-4 mm cartridge; flow rate: 1.0 mL/min; injection volume: 5.0 µL; detection at λ = 210 nm; solvents: A: water with 0.05% (v/v) trifluoroacetic acid; B: acetonitrile with 0.05% (v/v) trifluoroacetic acid: gradient elution: (A%): 0-4 min: 90%, 4-29 min: 90 $\rightarrow$ 0%, 29-31 min: 0%, 31-31.5 min: 0 $\rightarrow$ 90%, 31.5-40 min: 90%. The purity of all compounds was determined by this method.

Synthesis of 3

Compound **3** was synthesized starting from commercially available 7-methoxy-2,3,4,5-tetrahydro-1*H*-3-benzazepin-1-ol (**10**, Supplementary Figure 1) by removal of benzylic OH moiety to obtain **11**.[1] Cleavage of the methyl ether led to phenol **12**, which was converted into tertiary amine **3** by reductive alkylation with 4-phenylbutanal.[2]

7-Methoxy-2,3,4,5-tetrahydro-1*H*-3-benzazepine (11)[1]

Triethylsilane (3.3 mL, 20.7 mmol, 8.0 eq) and $BF_3 \cdot OEt_2$ (48 %, 2.7 mL, 10.4 mmol, 4.0 eq) were added to a solution of alcohol **10** (500 mg, 2.59 mmol, 1.0 eq) in CH_2Cl_2

(20 mL). The mixture was stirred for 4 h at rt. Then, 1 M NaOH (30 mL) was added, the organic layer was separated and the aqueous layer was extracted with CH_2Cl_2 (3 x 15 mL). The combined organic layers were dried (Na_2SO_4), filtered and concentrated *in vacuo*. The crude product was purified by fc (d = 3 cm, l = 20 cm, V = 12 mL, CH_2Cl_2:CH_3OH = 93:7 + 1 % *N*,*N*-dimethylethanamine). Pale yellow oil, yield 249 mg (1.40 mmol, 54 %). $C_{11}H_{15}NO$ (177.3). R_f = 0.32 (CH_2Cl_2:CH_3OH = 95:5 + 1 % *N*,*N*-dimethylethanamine). ^{1}H NMR (600 MHz, $CDCl_3$): δ [ppm] = 2.91-2.97 (m, 4H, 2 x 1-H, 2 x 5-H), 3.00-3.05 (m, 4H, 2 x 2-H, 2 x 4-H), 3.78 (s, 3H, OC*H*₃), 6.65 (dd, *J* = 8.2/2.7 Hz, 1H, 8-H), 6.68 (d, *J* = 2.6 Hz, 1H, 6-H), 7.02 (d, *J* = 8.2 Hz, 1H, 9-H). A signal for the NH proton is not seen in the spectrum. ^{13}C NMR (151 MHz, $CDCl_3$): δ [ppm] = 37.8 (1C, C-1), 38.9 (1C, C-5), 48.2 (1C, C-4), 48.6 (1C, C-2), 55.4 (1C, O*C*H₃), 110.9 (1C, C-8), 115.6 (1C, C-6), 130.4 (C-9), 133.8 (1C, C-9a), 142.9 (1C, C-5a), 158.2 (1C, C-7). HR-MS (APCI): m/z = 178.1215 (calcd. 178.1226 for $C_{11}H_{16}NO$ $[M+H]^+$). Purity (HPLC): 95.5 %, t_R = 11.0 min. IR: ṽ [cm^{-1}] = 3059 (C-H_{aryl}), 2947 (C-$H_{aliph.}$), 1605, 1582, 1501 (C=$C_{arom.}$), 1262 (C-O).

2,3,4,5-Tetrahydro-1*H*-3-benzazepin-7-ol (12)[2]

A solution of methyl ether **11** (240 mg, 1.35 mmol, 1.0 eq) in 48 % HBr (7 mL) was heated to reflux for 5 h. At rt, 1 M NaOH (20 mL) was added and the mixture was extracted with ethyl acetate (5 x 10 mL). The combined organic layers were dried (Na_2SO_4), filtered and concentrated *in vacuo*. The crude product was used for further reactions without purification. Pale yelow solid, mp 185 °C (decomp.), yield 192 mg (1.18 mmol, 87 %). $C_{10}H_{13}NO$ (163.2). R_f = 0.10 (CH_2Cl_2:CH_3OH = 85:15 + 1 % *N*,*N*-dimethylethanamine). ^{1}H NMR (400 MHz, CD_3OD): δ [ppm] = 2.79-2.93 (m, 8H, 2 x 1-H, 2 x 2-H, 2 x 4-H, 2 x 5-H), 6.51 (dd, *J* = 8.0/2.6 Hz, 1H, 8-H), 6.56 (d, *J* = 2.5 Hz, 1H, 6-H), 6.90 (d, *J* = 8.1 Hz, 1H, 9-H). Signals for the NH and OH protons are not seen in the spectrum. ^{13}C NMR (101 MHz, CD_3OD): δ [ppm] = 38.9 (1C, C-1), 39.9 (1C, C-5), 49.4 (1C, C-2 or C-4), 49.7 (1C, C-2 or C-4), 113.5 (1C, C-8), 117.3 (1C, C-6), 131.2 (C-9), 134.0 (1C, C-9a), 144.3 (1C, C-5a), 156.7 (1C, C-7). HR-MS (APCI): m/z = 164.1068 (calcd. 164.1070 for $C_{10}H_{14}NO$ $[M+H]^+$). Purity (HPLC): 90.4 %, t_R = 5.3 min. IR: ṽ [cm^{-1}] = 3020 (C-H_{aryl}), 2928 (C-$H_{aliph.}$), 1609, 1508 (C=$C_{arom.}$), 1462 (C-$H_{aliph.}$), 1258 (C-O).

3-(4-Phenylbutyl)-2,3,4,5-tetrahydro-1*H*-3-benzazepin-7-ol (3)

A mixture of secondary amine **3** (80.0 mg, 0.49 mmol, 1.1 eq), 4-phenylbutanal (68.5 mg, 0.46 mmol, 1.0 eq) and $NaBH(OAc)_3$ (148 mg, 0.70 mmol, 1.5 eq) in CH_2Cl_2 (5 mL) was stirred for 18 h at rt. A saturated solution of $NaHCO_3$ (10 mL) was added, the organic layer was separated and the aqueous layer was extracted with CH_2Cl_2 (3 x 10 mL). The combined organic layers were dried (Na_2SO_4), filtered and concentrated *in vacuo*. The crude product was purified by fc (d = 2 cm, l = 16 cm, V = 7 mL, cyclohexane:ethyl acetate = 6:4 + 1 % *N*,*N*-dimethylethanamine). Colorless solid, mp 123 °C, yield 92.3 mg (0.31 mmol, 68 %). $C_{20}H_{25}NO$ (295.4). R_f = 0.28 (cyclohexane:ethyl acetate = 1:1 + 1 % *N*,*N*-dimethylethanamine). ^{1}H NMR (400 MHz, CD_3OD): δ [ppm] = 1.51-1.66 (m, 4H, $NCH_2C\mathit{H_2}C\mathit{H_2}CH_2Ph$), 2.45-2.50 (m, 2H, $NC\mathit{H_2}CH_2CH_2CH_2Ph$), 2.53-2.65 (m, 6H, 2 x 2-H, 2 x 4-H, $NCH_2CH_2CH_2C\mathit{H_2}Ph$), 2.78-2.81 (m, 4H, 2 x 1-H, 2 x 5-H), 6.50 (dd, *J* = 8.0/2.6 Hz, 1H, 8-H), 6.54 (d, *J* = 2.5 Hz, 1H, 6-H), 6.87 (d, *J* = 8.0 Hz, 1H, 9-H), 7.12-7.19 (m, 3H, 2-H_{phenyl}, 4-H_{phenyl}, 6-H_{phenyl}), 7.22-7.27 (m, 2H, 3-H_{phenyl}, 5-H_{phenyl}). A signal for the OH proton is not seen in the spectrum. ^{13}C NMR (101 MHz, CD_3OD): δ [ppm] = 26.9 (1C, $NCH_2\mathit{C}H_2CH_2CH_2Ph$), 30.7 (1C, $NCH_2CH_2\mathit{C}H_2CH_2Ph$), 35.5 (1C, C-1), 36.5 (1C, C-5), 36.7 (1C, $NCH_2CH_2CH_2\mathit{C}H_2Ph$), 56.6 (1C, C-2 or C-4), 57.1 (1C, C-2 or C-4), 60.0 (1C, $N\mathit{C}H_2CH_2CH_2CH_2Ph$), 113.6 (1C, C-8), 116.9 (1C, C-6), 126.8 (1C, C-4_{phenyl}), 129.3 (2C, C-3_{phenyl}, C-5_{phenyl}), 129.4 (2C, C-2_{phenyl}, C-6_{phenyl}), 130.8 (1C, C-9), 133.8 (1C, C-9a), 143.6 (1C, C-1_{phenyl}), 144.1 (1C, C-5a), 156.8 (1C, C-7). HR-MS (APCI): m/z = 296.1989 (calcd. 296.2009 for $C_{20}H_{26}NO$ $[M+H]^+$). Purity (HPLC): 97.9 %, t_R = 16.7 min. IR: ṽ [cm^{-1}] = 3028 (C-H_{aryl}), 2924 (C-$H_{aliph.}$), 1605, 1493 (C=$C_{arom.}$), 1447 (C-$H_{aliph.}$), 1238 (C-O).

Supplementary References

1. Kanao, M. *et al.* Spasmolytic agents. I. Aminoalcohol esters having a phenethylamine-like moiety. *Chem. Pharm. Bull. (Tokyo).* **30,** 180–8 (1982).
2. Austin, N. E. *et al.* Novel 2,3,4,5-tetrahydro-1H-3-benzazepines with high affinity and selectivity for the dopamine D3 receptor. *Bioorg. Med. Chem. Lett.* **10,** 2553–5 (2000).
